# Sub-acute transverse colon volvulus an exceptional cause of large bowel obstruction: Case report

**DOI:** 10.1016/j.amsu.2021.01.102

**Published:** 2021-02-03

**Authors:** Abdelilah Elbakouri, Oussama Lafkih, Zineb Abbad El Andaloussi, Mounir Bouali, Khalid Elhattabi, Fatimazahra Bensardi, Abdelaziz Fadil

**Affiliations:** a Faculty of Medicine and Pharmacy, Hassan II University, Casablanca, Morocco; b Department of General Surgery, University Hospital Centre Ibn Rochd, Casablanca, Morocco; c Department of Radiology, University Hospital Centre Ibn Rochd, Casablanca, Morocco

**Keywords:** Volvulus, Transverse colon, Diagnosis, Surgery

## Abstract

**Introduction:**

The sub-acute form of transverse colon volvulus manifests with signs and symptoms of large bowel obstruction. The diagnosis is most often done intraoperatively. We report a rare case of transverse colon volvulus in a 65-year-old female patient with no particular pathological or surgical history.

**Case presentation:**

Sub-acute transverse colon volvulus in an elderly woman with no pathological or surgical history. Manifested with signs and symptoms of colonic obstruction. Surgically treated by a two-stage procedure with good postoperative outcomes.

**Discussion:**

The transverse colon volvulus represents only 2–4% of all colonic volvulus. We discuss the diagnostic and therapeutic approach of our case of transverse colon volvulus through a literature review.

**Conclusion:**

Transverse colon volvulus should be considered as a differential diagnosis in the face of large bowel obstruction. Early diagnosis and treatment improve the prognosis.

## HIGHLIGHTS

∗ Transverse colon volvulus (TCV) is an exceptional cause of colonic obstruction.
∗ The (TCV) diagnosis is difficult. It is most often done intraoperatively.
∗ The diagnosis delay can lead to necrosis and peritonitis.

## 1 Introduction

Transverse colon volvulus (TCV) is an exceptional cause of colonic obstruction with an important mortality rate. Colonic volvulus represents only 3–5% of all cases of intestinal obstruction. Among them, the transverse colon volvulus represent only 2–4% [1]. In its sub-acute form, it manifests with signs and symptoms of large bowel obstruction, but the diagnosis delay can lead to necrosis and peritonitis. The (TCV) diagnosis is difficult. It is most often done intraoperatively [2]. Which presents a real challenge for surgeons. We report a rare case of transverse colon volvulus in a 65-year-old female patient with no particular pathological or surgical history correlated with the visceral surgical emergency department of the IBN ROCHD university hospital center of Casablanca. This case report has been reported in line with the SCARE Criteria [3].

## 2 Case presentation

**Fig. 1:**
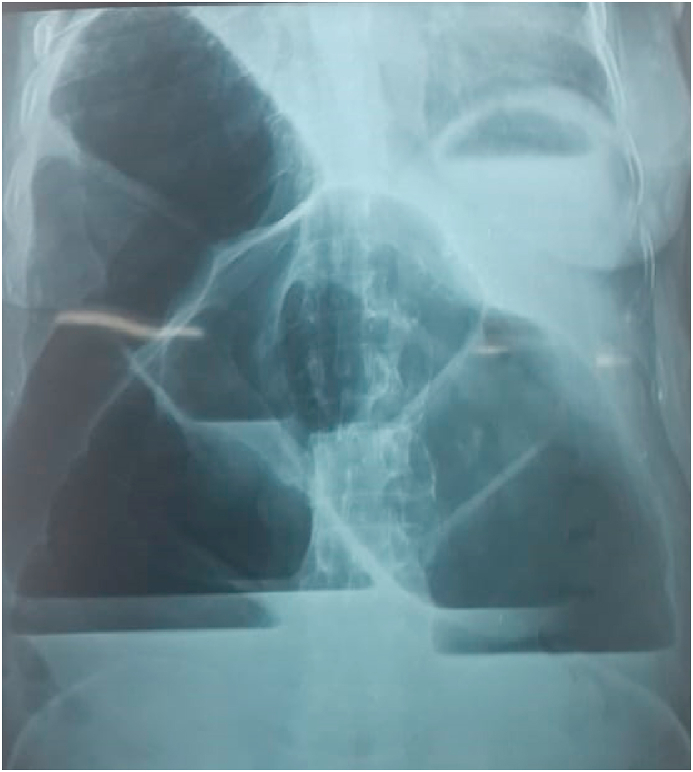
Plain abdominal X-ray showing the presence of a distended colon with two levels of fluid in the epigastrium.

**Fig. 2:**
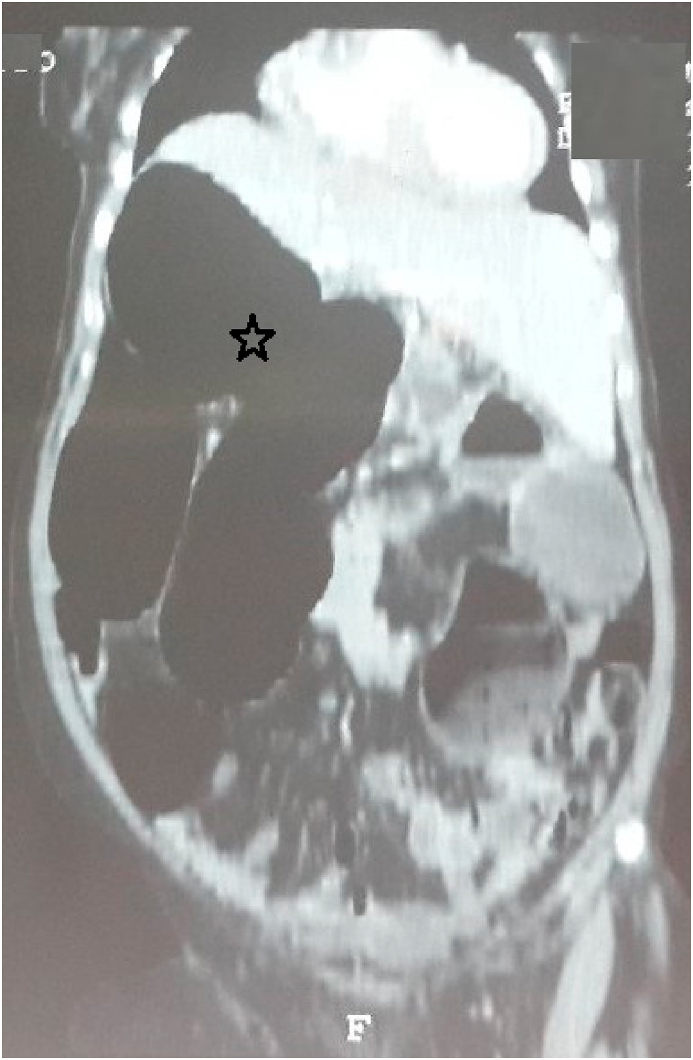
CT scan of the abdomen (coronal plane) showing a “U-shaped” loop of dilated colon (star) in the right upper abdomen.

**Fig. 3:**
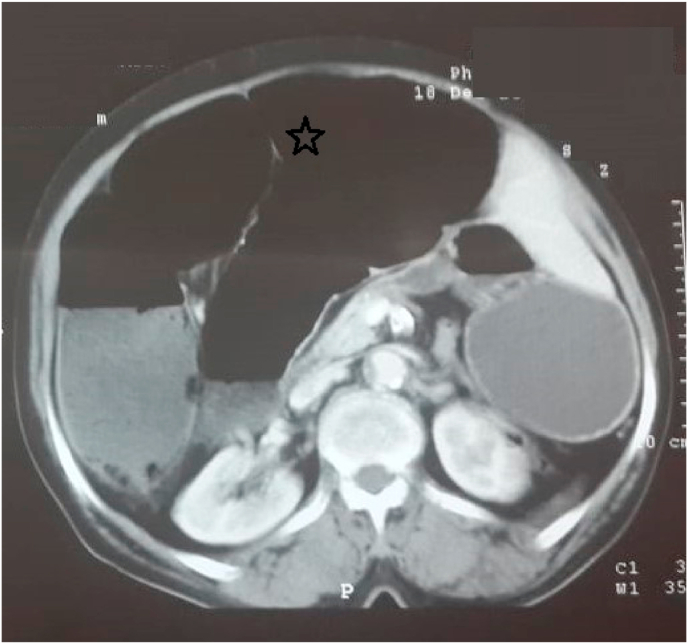
Axial CT image showing a “U-shaped” loop of dilated colon (star).

**Fig. 4:**
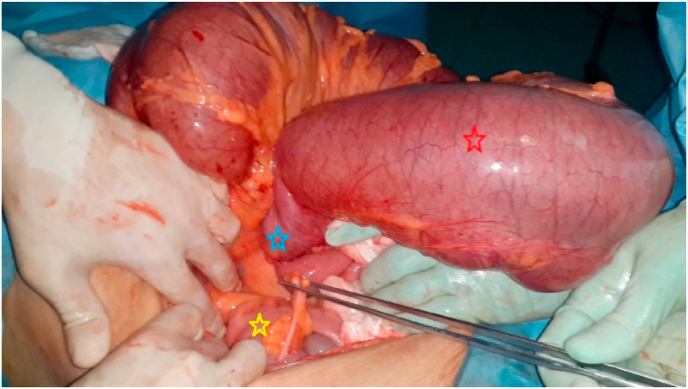
Dilated transverse colon volvulus twisted (red star) in clockwise direction on its mesentery. The point of twist was found in the right upper quadrant (blue star). The cecum was in normal position (yellow star). (For interpretation of the references to colour in this figure legend, the reader is referred to the Web version of this article.)

A 65-year-old woman, referred by family physician, to emergency with a 5-day history of constipation, important abdominal distension, nausea, vomiting and abdominal pain. Her last bowel movement had been 2 days ago. There was no significant past medical history or abdominal surgery. She had no family pathological, toxic or social history. On examination, his vital signs were: temperature 37.3 °C, pulse 107/minute, respiratory rate 25/minute, and blood pressure 110/60 mmHg. An abdominal examination revealed a massive distension of his abdomen without signs of peritonitis. His abdomen was tympanic to percussion. There were no umbilical or groin hernias. A digital rectal examination demonstrated an empty rectum. At this stage, the diagnosis of a strangulated large bowel obstruction was considered. An abdominal X-ray revealed a large bowel obstruction with two levels of fluid and a “U-shaped” loop in the right upper abdomen (Fig. 1). An abdominal CT scan showed a “U-shaped” loop of dilated colon (Fig. 2, Fig. 3). Correlating the clinical and imaging, the diagnosis of colonic obstruction by volvulus was the top of differential. The difficulty was to specify the incriminated colon segment. After optimization of his general condition with a Naso-Gastric tube suction and intravenous fluids resuscitation, a decision was taken to proceed with an emergency laparotomy, under general anesthesia with endotracheal intubation. Performed by an assistant professor, a 5th year and a 4th year surgical resident. Preoperative prophylactic antibiotics were administered. Intraoperative findings (Fig. 4) were of a dilated transverse colon volvulus twisted in a 360° clockwise direction on its mesentery. The point of twist was found in the right upper quadrant. The bowel was without signs of necrosis. The patient underwent transverse colectomy with Bouilly-Volkmann colostomy type. The postoperative course was uneventful: improvement of the general condition of the patient, colostomy was functional in the second post-operative day. He received proton pomp inhibitors (OMEPRAZOL 40 mg, one injection/day) and prophylactic heparin therapy (ENOXAPARIN SODIUM 0,4 ml = 40mg, one injection/day) and was discharged from the hospital four days later. A restoration of colonic continuity was performed 4 weeks after, which took place without complications. The patient was seen in two follow-up visits at 2 weeks and 4 weeks post-surgery with a satisfied recovery. He regained his normal activity.

## 3 Discussion

The first case of transverse colon volvulus to appear in the surgical literature was reported by the Finnish surgeon “Kallio” in 1932 [4]. The origin of the word “volvulus” comes from the Latin verb “volvere” which means “to turn”. It describes the abnormal torsion of the bowl. This torsion along the vascular axis (mesocolon) of the colon, resulting in venous and possibly arterial compromise, leading to intestinal ischemia and necrosis, causing colonic perforation and stercoral peritonitis [5].

The physio-pathological mechanism of volvulus formation is based on two properties: redundancy and non-fixation [6]. Like the sigmoid, the transverse colon is a mobile portion in the peritoneum appended by its mesocolon transversum. This allows the formation of a volvulus at this location [7]. risk factors that predispose to the onset of (TCV) are history of volvulus, previous abdominal surgery, congenital malformations such as intestinal malrotation with inadequate fixation of the posterior abdominal wall, Chilaiditis syndrome, pseudomembranous colitis associated with *Clostridium difficile*, pregnancy, and chronic constipation as it causes severe distension of the transverse colon [2].

There are two clinical forms of transverse colonic volvulus. The sub-acute form is characterized by a mild clinical picture due to the absence of ischemia in the early stages. It manifests as massive abdominal distension, mild abdominal pain without signs of peritoneal irritation. The white blood cell count is often normal or slightly increased. This form usually occurs in elderly bedridden patients with multiple comorbidities. Our patient was in good general condition without any associated pathology. The diagnosis and treatment delay can cause progression to the acute fulminant form [1,8,9]. Other type, the acute fulminant form, present with signs of bowel necrosis manifested by an acute abdomen clinical picture, made of severe abdominal pain with generalized rebound tenderness, but less abdominal distension than in the subacute form, with marked leukocytosis, which presents a surgical emergency to resect the compromised bowl [[8], [9], [10]]. The diagnosis of transverse colon volvulus is most often made intraoperatively because the radiographic findings are not as characteristic. There are no pathognomonic radiographic signs. The X-ray reveal the presence of a distended colon with two levels of fluid in the epigastrium. In the subacute type, the achievement of an early diagnosis through abdominal CT scan is advised, but it is not recommended in acute settings because it can delay surgical intervention [2,4]. The CT scan findings in our case suggested a volvulus, without being able to identify its exact site, but the diagnostic confirmation was only made intraoperatively.

Transverse colon volvulus is a surgical emergency. Resection of the involved segment constitutes the treatment of choice [11]. Followed by primary anastomosis in a one-stage procedure. Or a stoma is created; in a two-stage procedure; 2–3 months post-surgery, end-to-end anastomosis is performed. The choice of a procedure depends on the general condition of the patient, the presence or not of peritonitis and the local condition of the colon [5]. In our case we chose the second option (two-stage procedure) given the general state of the patient who presented hemodynamic instability during the operation. In the presence of megacolon some authors suggest performing a sub-total colectomy [12].

## 4 Conclusion

Transverse colon volvulus is an exceptional condition with high mortality rate. It should be considered as a differential diagnosis in the face of large bowel obstruction. In the subacute form, the achievement of an early diagnosis through abdominal CT scan is advised, which should not delay surgical management, in order to improve the prognosis. The resection of the segment involved is the treatment of choice.

## Sources of funding

No sources of funding.

## Ethical approval

I declare on my honor that the ethical approval has been exempted by my establishment.

## Consent written

Informed consent was obtained from the patient for publication of this case report and accompanying images. A copy of the written consent is available for review by the Editor-in-Chief of this journal on request.

## Registration of research

This is a case report.

## Provenance and peer review

Not commissioned, externally peer-reviewed.

## Annals of medicine and surgery

The following information is required for submission. Please note that failure to respond to these questions/statements will mean your submission will be returned. If you have nothing to declare in any of these categories then this should be stated.

## Please state any conflicts of interest

The authors declare having no conflicts of interest for this article.

## Please state any sources of funding for your research

No sources of funding.

## Ethical approval

I declare on my honour that the ethical approval has been exempted by my establishment.

## Consent

Informed consent was obtained from the patient for publication of this case report and accompanying images. A copy of the written consent is available for review by the Editor-in-Chief of this journal on request.

## Author contribution

Oussama Lafkih: Corresponding author writing the paper, Abdelilah Elbakouri: writing the paper, Zineb Abbad El Andaloussi: writing the paper, Mounir Bouali: study concept, Khalid Elhattabi: correction of the paper, Fatimazahra Bensardi: correction of the paper, Abdelaziz Fadil: correction of the paper.

## Registration of research studies

This is a case report.

## Guarantor

Dr. Oussama Lafkih

## Declaration of competing interest

The authors declare having no conflicts of interest for this article.
